# Detecting Vital Signs with Wearable Wireless Sensors

**DOI:** 10.3390/s101210837

**Published:** 2010-12-02

**Authors:** Tuba Yilmaz, Robert Foster, Yang Hao

**Affiliations:** Department of Electronic Engineering, Queen Mary University of London, Mile End Road, London, E1 4NS, UK; E-Mail: tuba.yilmaz@elec.qmul.ac.uk(T.Y.)

**Keywords:** on-body sensors, wearable sensors, RF sensing, wireless telemetry, BAN networks

## Abstract

The emergence of wireless technologies and advancements in on-body sensor design can enable change in the conventional health-care system, replacing it with wearable health-care systems, centred on the individual. Wearable monitoring systems can provide continuous physiological data, as well as better information regarding the general health of individuals. Thus, such vital-sign monitoring systems will reduce health-care costs by disease prevention and enhance the quality of life with disease management. In this paper, recent progress in non-invasive monitoring technologies for chronic disease management is reviewed. In particular, devices and techniques for monitoring blood pressure, blood glucose levels, cardiac activity and respiratory activity are discussed; in addition, on-body propagation issues for multiple sensors are presented.

## 1. Introduction

The design of wearable physiological measurement systems has been a growing research interest in the last decade, due to the potential applications in medicine, sports and security. With the increase in the size of the elderly population, as well as the emergence of chronic diseases because of the changes in lifestyle, there has been a need to monitor the health status of individuals in their daily routine to prevent fatal disorders. The adoption of mobile health-care technology is promising to enhance the quality of life for chronic disease patients and the elderly, as well as healthy individuals. Furthermore, it offers the potential to alter the current heath-care system by enabling out-patient care and preventing unnecessary hospitalisations. Designing a telemetry system for health monitoring is a very cumbersome task. There are many key issues to be addressed, including:

- designing reliable sensors;
- ensuring the reliable transmission of vital sign data;
- providing privacy and security for individuals.

Mobility is both a key benefit of such systems and a constraint on their design. To achieve this benefit, wireless physiological sensors must be small, low-weight, low-power and, of course, wireless. The radio channel on and around the body has particular challenges not found elsewhere. For instance, antennas designed to operate at a given frequency in free space will operate at a lower frequency when placed on the body—so-called ‘de-tuning’ [1]. Losses are increased on-body compared with free space; there are also shadowing issues and an especially challenging dynamic aspect to all these problems, due to the variations in posture and movement that occur.

Although an extensive literature review on mobile healthcare is beyond the scope of this paper, a detailed review of recent publications is given on research on chronic disease management and on-body propagation issues. The remainder of this paper is divided into two main sections; the first examines recent research on the measurement of the major physiological parameters, termed ‘vital signs’. The second examines wearable wireless sensors from an electromagnetic perspective, discussing such issues as communications protocols, antenna design and radio channel propagation on and around the body.

## 2. Vital Sign Monitoring

There are a number of health issues whose treatment benefits from continuous vital sign monitoring. Traditionally, when this approach is deemed necessary, it results in the hospitalisation of the patient, with expensive equipment and medical personnel on hand; in some cases, the patient may remain at home, but the use of bulky and expensive equipment remains. Much effort has gone into the development of small, wearable devices over recent years, with benefits including lower cost, greater mobility for the patient and, potentially, improved physiological data for the physicians to analyse when attempting to diagnose the condition. Wirelessly-enabling these devices provides greater mobility and improves the efficiency with which available bandwidth is used [2]. A review of wireless body sensor networks (WBSNs) for health-care applications was given in [2], including a design strategy for such wireless sensors.

There are a number of demographic changes to world populations, particularly in the West, that are driving the move towards use of WBSNs and vital sign monitoring. The two most significant changes are the ageing of the population and the rise in obesity levels. Both of these factors increase the risk of developing various conditions that require significant medical intervention and, thus, significant cost. Governments around the world have acknowledged this fact and are seeking ways of delivering healthcare more efficiently, including the use of ‘personal health systems’ and tele-medicine techniques.

The two conditions of most interest currently are diabetes and cardiovascular disease and its related conditions. In developed countries, typically 24% of the population suffers from diabetes and the further complications of the disease, such as cardiovascular disease, can only be prevented with frequent monitoring of blood glucose levels (BGL) [3].

Hypertension is another common health problem which has no obvious symptoms. Healthy dietary choices, lifestyle changes and use of medicine can improve the condition of the patient. In order to control the risks associated with the disease, it is crucial to monitor the blood pressure (BP) of the patient frequently. For middle-aged and older subjects, high blood pressure is an important indicator for cardiovascular diseases, which are amongst the leading cause of sudden deaths globally.

Vital sign monitoring systems will, therefore, involve monitoring one or more of the following:

- blood glucose level;
- blood pressure;
- pulse rate;
- electrocardiograph (ECG) patterns;
- respiration rate;
- respiration effectiveness (e.g., blood oxygen saturation).

These are discussed in more detail in the following sections.

### 2.1. Monitoring Blood Pressure

Traditionally, monitoring of blood pressure is performed in a clinic with trained personnel by mounting inflatable pressure cuffs with stethoscopes to the patient’s arm—the so-called auscultatory method. This type of measurement does not achieve continuous monitoring and the patient is required to be in a certain posture in the clinical environment, which causes the *white-coat effect*. The white-coat effect is known to be the falsification of BP measurements, usually with transient peaks in BP, due to the stress caused by being present in a clinical environment [4]. Current alternatives are ambulatory monitoring and home-monitoring devices, where patients can monitor their own BP.

One of the common techniques used in ambulatory/home monitoring devices is the oscillometric method. Home monitoring devices generally include a fully automatic inflatable cuff which can be mounted on the patient’s wrist and measures the blood pressure of the radial artery by relating external pressure with the magnitude of arterial volume pulsations.

Although oscillometric monitoring systems are convenient to avoid the transient rise of BP due to the white-coat effect, the periodic interruptions of the blood flow and continuous usage of such devices may cause unwanted side effects, such as sleep disruptions at night-time, skin irritations and an increase in stress levels [5]. Vasotrac (Medwave Inc., Arden Hills, MN) is an example of a watch-type ambulatory non-invasive BP monitoring device [6,7]. It includes a circular sensor (this must sit on top of the radial artery in the wrist), a digital monitor and a disposable adhesive plaster to estimate the location of artery. After collection of data, the sensor response is taken and processed by a controller unit and displayed on the digital screen. Although Vasotrac is a cuff-less device, an external pressure is required for reliable measurements. Vasotrac is a good alternative for infrequent ambulatory monitoring; it is, however, unsuitable for continuous monitoring.

Many different methods have been used to measure BP non-invasively. For example, the ultrasonic method, introduced in 1961, relates the Doppler shift of the ultrasound response and the velocity of blood [8]. The frequency shift of the scattered ultrasound with the change in the velocity of the particles in blood is observed by placing two piezoelectric crystals in a plastic tube. The Doppler shift technique is proven to be an effective method for measuring the systolic BP of infants [9].

Measuring the BP through pulse wave transit time (PTT) is another cuff-less technique and a candidate method for continuous monitoring of BP [10]. When measuring the PTT, the heart activity is usually monitored with an ECG sensor and a photoplethysmogram (PPG) sensor is placed on a finger, wrist or earlobe to track the pulse travelling from the heart to the peripheral point. Simply, if the arterial pressure is higher, the pulse travels faster. Recently, a wrist module was developed to measure BP by integrating a PPG sensor and ECG sensor into a watch-type monitoring device [11,12]. However, the reliability of measurements and calibration of the device are still issues under investigation.

**Figure 1.**
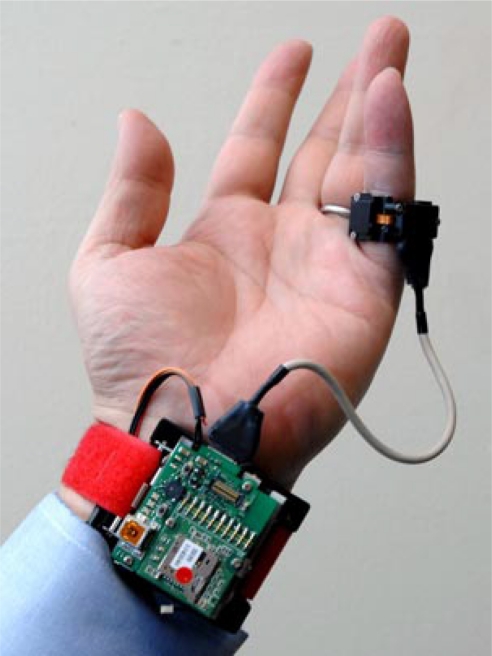
Blood pressure sensor by MIT [15].

More recently, another cuff-less design was developed by using combined PTT and oscillometric methods [13–15]. The system estimates the BP by placing two sensors along the artery; typically, these are at the wrist and index finger, as shown in Figure 1. However, measuring the blood pressure in this fashion is challenging, due to instabilities in hydrostatic pressure caused by the change of hand position with respect to the heart. Conventional ambulatory BP meters require patients to sit and raise their hand to the heart level. In order to overcome this challenge, a MEMS accelerometer is used to adjust the height of the hand with respect to the heart, to set the hydrostatic pressure offset for the PTT sensor. This approach allows the patients move their hands freely by calibrating the PTT sensor response according to the change in hydrostatic pressure. One benefit is that the local pressure applied to the tissue is trivial compared to the traditional oscillometric devices and does not interrupt the blood flow. Thus, this design is a promising approach to achieve continuous, non-invasive and unobtrusive monitoring of blood pressure and is one of the cutting-edge technologies under investigation at this time.

### 2.2. Monitoring the Blood Glucose Levels

Most commercial blood glucose (BG) monitoring devices employ invasive techniques; usually, a blood sample must be obtained by pricking the finger with a lancet. The blood sample obtained is then exposed to a strip and the BGL calculated by inserting the strip into a digital monitor. Diabetic patients should perform the task at least 5–6 times a day for tight metabolic control. However, the finger pricking task is reported to be a painful procedure, leading some to take fewer samples, hence risking problems induced by poor BGL management.

Some commercial systems (e.g., Medtronic’s MiniMed and Guardian products [16]) are termed ‘minimally-invasive’ continuous monitoring systems. Typically, a (disposable) bio-sensor needle is inserted under the skin on the abdomen and the BGL is derived from the glucose level in the interstitial tissue fluid. In Medtronic’s product [16], the needle unit is self-contained and includes all electronics required to capture the signal, process it and communicate it wirelessly to a second body-worn unit that acts as the user-interface. Recently, the authors were involved in a feasibility study that examined a similar system concept, with the aim of extending the lifetime of the bio-sensor needle [17]. This involved both design and optimisation of the bio-sensor and the use of signal processing techniques to extract the maximum useful information possible. Work is on-going in this area.

A non-invasive continuous self-monitoring system is key to improve the management of diabetes. In recent years, there has been a considerable progress in research on minimally-invasive or non-invasive monitoring techniques. One example is the GlucoWatch Biographer, a minimally-invasive device measuring BGL through extraction of transdermal fluid. This device was developed by Cygnus, Inc., and was commercially available for some time [18]. However, the Food and Drug Administration (FDA) in the United States later banned the commercial use of the device, due to complaints from users claiming that the device caused a mild-to-moderate amount of skin irritation. The device essentially drew the interstitial fluid through the skin with a low electrical current; therefore, there was a 10–15 minutes time lag compared to traditional glucometers.

**Figure 2.**
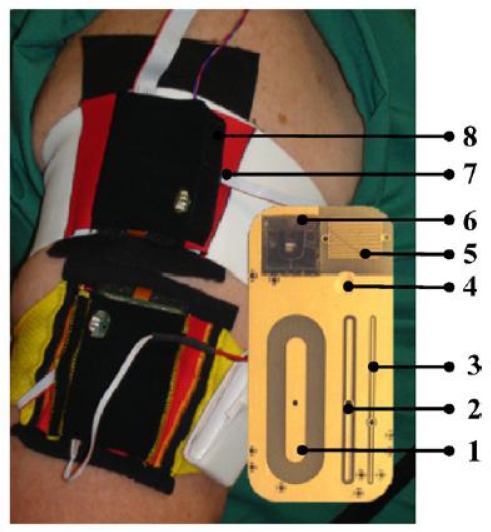
Arm module proposed in [23] (1) deep, (2) mid and (3) shallow electrodes. (4) Temperature sensor. (5) Sweat sensor. (6) Siliconwafer based optical reflection sensor. (7) Humidity sensor. (8) 3-axes acceleration sensor.

Impedance spectroscopy (IS) is another highly investigated method; it measures the change in the electrical properties of blood non-invasively. Variation in BGL affects the electrical properties of erythrocyte (red blood cell) membranes; this causes alterations in the electrolyte balance of skin and subcutaneous tissue [19,20]. These variations can be detected with the IS technique by measuring the magnitude of impedance *|Z|* with an RLC resonant circuit [21] or a vector network analyser (VNA). Although IS is sensitive to BG variation, the alterations in other bodily parameters (such as sweat levels, changes in the posture and temperature levels) can affect the measurement results. In order to reduce the effect of the other parameters, a multi-sensor approach has recently been developed [22,23]. The new arm module, shown in Figure 2, includes eight sensors; three of them are capacitive electrodes in long, medium and short lengths and operating at different frequencies, developed by Solianis Monitoring AG. The medium-length and long electrodes penetrate to deeper layers of tissue, providing information related to changes in glucose levels; the short electrode penetrates to the surface layer of skin, providing data related with to other parameters. Additionally, there is a sweat sensor, an accelerometer and an optical reflection sensor, which are combined in an arm module on elasticized cloth. The arm module was tested under controlled conditions with ten male diabetic patients; however, further investigation is crucial to understand the true performance of the developed module in non-laboratory environments.

It is well-known that alterations in BGL affects the electrical properties of blood, as well as the electrical properties of subcutaneous tissue, at microwave frequencies. In order to measure the change in the electrical properties of interstitial fluid with respect to glucose levels, an *in vitro* study performed by collecting the blood plasmas of twelve healthy volunteers [24]. The electrical properties of blood plasma were measured between 100 MHz and 20 GHz using Agilent’s open-ended slim coaxial probe kit; this was performed by adding dextrose to the plasma sample in concentration from 0 mg/dL to 16,000 mg/dL. According to the measured results, the conductivity values between 15 GHz and 20 GHz were more sensitive to changes in the glucose concentrations in the blood plasma.

Another experiment was performed to relate the change in microwave resonance characteristics with the alterations in BGL [25,26]. A blood sample containing 6 mmol/dl of glucose was poured in a cubic test cell and exposed to low power microwave energy between 10 GHz to 20 GHz. This was achieved by placing two SMA co-axial-to-waveguide 18 (WR62) E-field adaptors on each side of the test cell. The blood sample was switched with another blood sample containing 14 mmol/dL glucose; for both cases, the *S*_21_ magnitude response of the system (*i.e.*, the transmission coefficient or transfer function) was measured with an Agilent 8720ET VNA. The observed minimum in the *S*_21_ response for the first sample occurred at 13.130 GHz; for the second test sample, the frequency of the minimum shifted upwards by 332 MHz, occurring at 13.452 GHz. The frequency shift occurs due to the permittivity change in the blood glucose levels. Also, it was observed that the Q factor (a measure of the bandwidth and loss in a system) for the first sample was higher than for the second sample. Both experiments showed that microwave detection is a promising approach to detect blood glucose levels non-invasively. However, *in vivo* measurements of electrical properties, as well as the effect of other parameters in blood on the electrical properties, should be further investigated.

**Figure 3.**
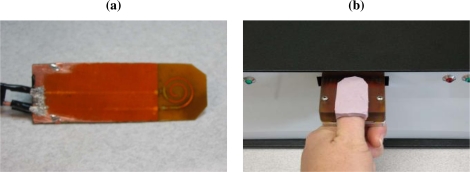
(a) Initial design of microwave sensor (b) Modified microwave sensor with silicon positioning aid [27].

Recently, a spiral-shaped microwave sensor, shown in Figure 3, has been developed and tested for non-invasive monitoring of BGL [27]. The sensor was designed by using the microstrip ring-resonator method. Testing of the sensor was performed with a ‘soda test’, in which the author fasted for at least eight hours before consuming a soda drink with high sugar content to increase the BGL rapidly. During the soda test, the author measured the sensor response for two hours at ten-minute intervals, by placing the radiating part against the wrist. Meanwhile, the blood glucose level was tracked with a commercial glucometer. The first maximum of the *S*_21_ response was tracked. The change in sensor response was correlated with the measured BGL. Although this approach showed promising results, the measurements should also be performed in a less-controlled (*i.e.*, more realistic) environment with more subjects. The designed sensor is still bulky; in addition, the response of the sensor also changes with the applied pressure. In future designs, the sensor should be integrated in a more flexible structure with controlled pressure.

Another preliminary study was published recently on non-invasive measurement of BGL using millimetre-waves [28,29]. In the study, a metal fibre was connected to a waveguide and the return loss of the system measured by placing different glucose solutions as a load. Although the change in the frequency of resonance, as well as the Q factor, is in an acceptable range, human tissue is, in reality, very lossy; therefore, the penetration depth at higher frequencies will be very low. This particular problem has been addressed in the detection of BGL with Raman spectroscopy.

Usually, in near-IR spectroscopy, the peripheral tissue is exposed to the near-IR radiation and the transmission or reflection from the tissue is measured. The blood glucose data is extracted through analysis of the measured tissue response. In [30], the validity of the extracted glucose values from measured spectral absorption was discussed. The absorption of the radiation by glucose is very small, compared with absorption from background tissues. Experiments were performed using a protein solution with beef fat; under controlled conditions, the near-IR measurements were taken whilst altering the glucose levels. It was observed that, although no glucose was present, the measurement results was predicting its presence. Recently, algorithms for better estimation of the blood analytes were developed using Raman spectroscopy [31]. ([30,31] contain a good overview of the challenges that must be overcome by spectroscopic methods of determining BGL for the interested reader.) Turbidity is one of the major causes of intensity and shape distortions in Raman spectra. The proposed method corrects intensity and shape distortions of the Raman spectra; it was tested with a set of tissue phantoms with same concentration of Raman scatterers but with different background turbidities. This method presents a promising approach on correcting intrinsic line shapes and intensity information. More recently, an algorithm was developed to estimate the BGL change from the change in the glucose levels in transcutaneous tissue [32]. However, alterations in the glucose level of interstitial fluid lags behind the change in BGL. This time lag should be addressed and estimation should be performed accordingly.

### 2.3. Monitoring the Cardiac Activity

Conventional monitoring of cardiac activity is performed in a clinical setting in real-time during a visit to the facility, by recording electrocardiograph (ECG) signals. Monitoring the heart activity through ECG signals is a very common technique, performed by placing at least three electrodes to the skin to measure the electrical activity of heart. Traditionally, Holter monitors are used for ambulatory monitoring during the recovery period after cardiac surgeries [33]. Although Holter monitors are capable of providing continuous monitoring, the central unit of these monitors is bulky and each electrode is connected to the central unit with wires. Therefore, use of the Holter monitor interrupts the daily routine of the patient and is not feasible for unobtrusive continuous monitoring. Over the past few years, with the advancement in wireless technologies, Holter monitors have been miniaturized and evolved into complete wire-free monitoring devices. Although ambulatory wire-free devices look promising for continuous monitoring, there is still a need for further development of such devices.

Development of an ideal electrode for ambulatory devices is vital to achieve continuous unobtrusive monitoring. Commonly-used electrodes for clinical applications are gel-type Ag/AgCl wet electrodes. Even though these electrodes have been reliable, compact and low-cost, continuous usage of wet electrodes causes skin irritations, since these electrodes employs conductive adhesives in order to maintain the resistive electrical contact with the skin. Also, the signal quality from these electrodes decreases significantly the gel dries, due to the loss of proper contact with the skin. As an alternative to wet electrodes, dry electrodes were developed; however, most dry electrodes are not bio-compatible, since they are usually constructed with hard substrates. Also, when placed on skin, dry electrodes have a higher impedance than gel-type ones [34,35]. More flexible dry electrodes have been developed by using conductive rubber or elastic materials instead of hard substrates [36]. Conductive-rubber-based electrodes use human sweat to maintain the contact with the skin, instead of conductive gel. These electrodes can be integrated into clothing. As well as being flexible, the conductive rubber is approved for short-term implants; thus, it is expected to cause the least skin irritation. However, these electrodes still require contact with the skin and long-term usage of rubber-based dry electrodes still causes skin irritation. Motion is another factor that affects the measurement results: it causes a change in external pressure and eventually affects the contact between the electrode and skin.

**Figure 4.**
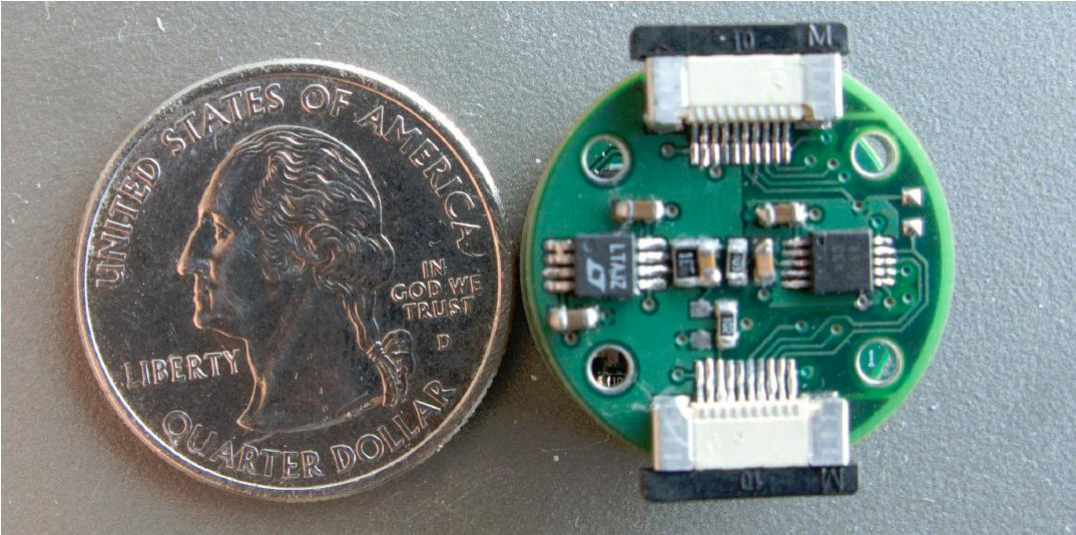
Capacitive electrodes proposed in [38].

Alternatively, insulated electrodes are capable of sensing ECG signals through clothing via capacitive sensing, without a resistive electrical contact with skin, as shown in Figure 4 [37,38]. Earlier versions of capacitive electrodes used uncomfortable materials in order to provide high capacitance. However, over the last few years, bio-compatible materials have been used and these electrodes have been integrated with small wireless sensing devices, termed ‘motes’. Additionally, capacitive electrodes provide good signal quality regardless of motion. Thus, insulated bio-electrodes are a promising approach for wireless cardiac monitoring and are an example of the current state-of-the-art technology in this field.

Mechanical cardiac activity can be tracked with microwave sensing. Microwave sensing does not require direct contact with the skin and employs a transmitter and a receiver in which the exact position of chest is detected by demodulating the phase of a scattered Doppler radar wave. Recently, a single-chip implementation of a Doppler radar technique has been presented [39]. However, a detailed morphology of the ECG is not available with the Doppler radar technique.

### 2.4. Monitoring Respiration

In clinical research, conventional non-invasive monitoring of respiration rate is performed by impedance pneumography and inductive plethysmography. Impedance pneumography measures the impedance change between two electrodes placed on the chest. Thus, this technique measures the movement of the chest caused by the respiration cycle. Impedance pneumography is prone to errors from posture changes and motion. Inductive plethysmography, on the other hand, employs two copper wires: one is placed around the abdomen, the other placed on the chest. During the respiration cycle, volumetric differences occur and this causes self-induction of the two wires. Inductive plethysmography is a more reliable technique compared to impedance pneumography.

Recently, a measurement system for drivers was introduced, where the respiration rate is derived by measuring the pressure applied to a gauge embedded in a seat belt [40]. Alternatively, a yarn-based piezo-resistive textile sensor has been developed to estimates the respiration rate through the strain output of the sensor when it is subjected to tensile strength [41]. Another study compares the measurements of heart rate and respiration cycle outputs of two different materials: one piezoelectric plastic polyvinylidenefluoride (PVDF) and the other electromechanical film (EMFi) embedded into clothing [42]. The PVDF sensor produces electrical signals with the mechanical changes in material. The EMFi sensor has two electrodes embedded in fabric and connected to each other with conductive wires. Although this study found similar results between the two sensors, the study was performed when the subjects were in a resting state. Other non-invasive measurement techniques include estimating the respiration rates through cardiac activity.

### 2.5. Multi-Parameter Monitoring

Ideally, monitoring systems for mobile health should be unobtrusive, incorporate multiple sensors, give real-time feedback and provide wireless communication with on-line data evaluation. To this end, several papers have been published and commercial multi-parameter sensors have also been produced. Of these commercial products, devices for performance monitoring (such as the Nike iPod kit and ‘adidas miCoach’) have been successful. However, these devices are limited to measuring one or two parameters and are not suitable for monitoring chronic disease patients. Combining sensors for vital sign monitoring has been studied by research groups in academia, as well as in industry. LifeShirt (Vivometrics Inc., USA) is an example of a multi-parameter monitoring system [43]. LifeShirt includes a garment, analysis software and a data recorder. Respiration, ECG, activity and posture are monitored with the sensors attached to the garment. The device has been tested in many clinical settings and has been used successfully in animal studies as well. Even though LifeShirt is a promising invention, it is lacking wireless nodes to provide real-time data transfer. ‘WEALTHY’ is another textile-based garment developed to monitor ECG, respiration, activity and temperature measurements [44]. ECG monitoring is performed with knitted yarn-based sensors integrated into the wearable garment. When the patient is in a resting state, good quality signals are obtained; however, during physical activity, the movement of the arm causes significant noise. Therefore, hydrogel is required to maintain the ohmic connection with the skin. The system employs piezoresistive sensors for respiration and activity sensing. The main goal of textile-based systems is to develop a wearable and washable garment for monitoring vital signs data; they are usually aimed at cardiac, asthma and sleep apnea patients.

**Figure 5.**
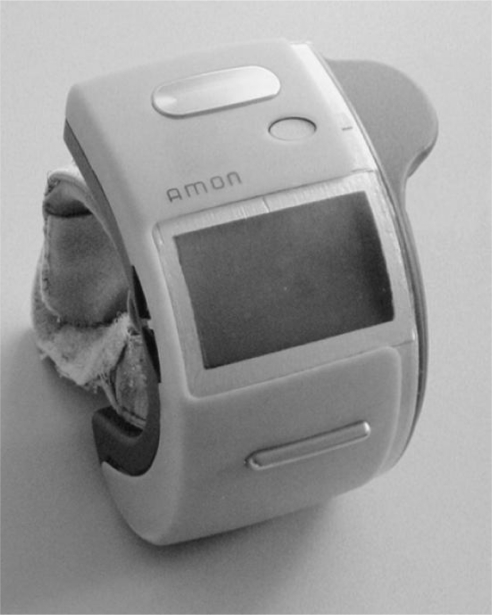
AMON wrist module [46].

Another study on multiple health monitoring devices is ‘AMON’, shown in Figure 5, which employs a miniaturized wrist-type device and a stationary device [45,46]. AMON is capable of monitoring blood oxygen saturation (SPO2), temperature and activity continuously. It employs a reflectance sensor for SPO2 measurement. The reflectance sensor detects the SPO2 levels by measuring the absorbance levels of two different signals with two wavelengths. The activity of the individual is measured by acceleration sensors. Besides the aforementioned sensors, the wrist-type device also employs an oscillometric blood pressure sensor and single lead ECG monitoring. The ECG and blood pressure measurements are taken three times a day or upon the request of the patient. ECG monitoring is performed with gold electrodes (which has higher impedance) and is converted into a twelve lead ECG signal at the stationary unit; therefore, the reliability of the ECG results is questionable. Additionally, for blood pressure monitoring, AMON incorporates the traditional inflatable cuffs; thus, the mobility of the patient is restricted during the measurement. The advantage of the AMON system is that the system includes a mobile communication (GSM) transceiver, which enables data exchange with the health-care provider. Moreover, the device can perform data analysis on-line, enabling real-time feedback and emergency detection. Many other devices combining several sensors to monitor multiple parameters are available, such as SenseWear [47], Escort Guardian [48], Micropack [49], Smartshirt [50] and VTAMN [51]. These products and studies have been reviewed in the literature numerous times; thus, a detailed review of them is not included.

## 3. Wireless Technologies

Current physiological monitoring systems offer different means of communication between on-body sensors and the main data-capture unit. The simplest way of providing secure data transfer between the sensors and the main unit is wires. The implementation of wires in such systems is easy and low-cost; an obvious example of this technique is the aforementioned Holter monitor. However, these monitoring devices are obtrusive, presenting a challenge to patients in continuing their daily routine; in addition, the risk of wire tangling may mean that system failure is more likely. Over the past few decades, a number of alternative communication techniques have emerged. One is the use of so-called ‘smart clothes’ for on-body communications, where electronic devices (and, usually, interconnections in the form of wires) are embedded and woven into the fabrics. However, smart clothes are expensive and may force the end user to ignore personal preferences. Another innovative approach is communicating through biological channels (bio-channels); here, the human body is used as a communication channel by allowing data transmission between near electronic devices through near-field electrostatic coupling [52]. One limitation is that only very small amounts of data can be transmitted through bio-channels; therefore, their use is not desirable for body area networks (BANs).

There are three general scenarios for wireless body-centric communications [1]:

- *off-body*, where a device located on a body communicates with one or more devices located off-body;
- *on-body*, where a number of devices located on the body communicate with each other;
- *in-body*, where some (or all) of the devices on body are implanted, rather than worn (e.g., pace-makers).

The latter two cases can be grouped together under the term ‘*intra-body communications*’. Where the off-body case deals with communications between wireless BANs (WBANs), it is termed ‘*inter-body communications*’. It should be noted that the off-body case can cover propagation distances ranging from less than a metre up to tens of metres, whereas the intra-body cases will be less than two metres (the most extreme case could be a device near the foot communicating with a device near the head). Inter-operability of WBANs that may be located in close proximity is a critical issue. The off-body case is exemplified by mobile telephones; a great deal of research has examined how the performance of these devices is affected by proximity to the human body. This scenario will not be discussed further in this paper; the interested reader is directed to the literature (see, for example, [53–59]).

With the recent advancements in wireless technologies, wearable monitoring systems can operate without wires by integrating wireless modules with on-body sensors. Using wireless communication is beneficial in many ways. First and foremost, real-time monitoring of collected data can be achieved more easily, which is useful for launching alert mechanisms. In addition, wireless on-body sensors are more unobtrusive for patients, allowing them to continue with their daily routine more easily. Moreover, these systems enable out-patient care, potentially even after more significant operations, thus decreasing healthcare costs. Finally, by allowing the individuals to track their own data with real-time feedback through smart-phones or PDAs, chronic disease sufferers can manage their disease more efficiently.

Providing reliable wireless transmission of data between on-body sensors (and also off-body to mobile, or stationary, devices) is a demanding task. There are a number of fundamental challenges that exist in understanding the propagation characteristics around the body, as well as the means of coupling radiated energy into these propagation channels through optimum antenna design [1]. From a communications perspective, different applications may have different bandwidth requirements, whilst all physiological data must be treated in a careful and secure manner; these lead to certain constraints on the optimum communications protocol for WBAN applications. Furthermore, the wireless module should be light-weight, low-power, low-cost and compatible with the remaining circuitry [2]; these constraints will also inform protocol and antenna design. Recent progress in addressing these challenges is reviewed in the following sections.

Whilst simple one-sensor systems are valid examples of the WBAN concept, it should be noted that the greatest realisation of the potential of a WBAN system is when applied to multiple sensor nodes on-body, such as the multi-parameter monitoring systems discussed in Section 2.5.

### 3.1. Wireless Communications Protocols

Several approaches have been developed for on-body wireless communication; for example, IEEE 802.15.1 (Bluetooth) is one of the widely-used standards for wireless *Personal Area Networks* (WPANs)—these differ from WBANs in the extent of the propagation range: WBANs cover the immediate area of the body (intra-body communications), whilst WPANs extend away from the body (off-body communications). However, some of the uses overlap and are difficult to distinguish; a typical example of Bluetooth usage for WBANs is transmission of voice data between a head-set and a cell phone [60]. Bluetooth-enabled devices can operate at the unlicensed industrial, scientific, and medical (ISM) band around 2.45GHz (2.40–2.48 GHz), divided into 79 channels, with a 1 Mbps data rate. To minimise interference and fading, a frequency-hopping spread-spectrum technique (FHSS) is used. The modulation technique is Gaussian frequency-shift keying and it is capable of transmitting data to a range of between two to ten metres. The maximum transmitted power is 0 dBm (1 mW). Multiple Bluetooth devices can form a piconet, a star-topology network consisting of a master device communicating with seven slave devices. This is the basic configuration, although multiple piconets can be linked into ‘scatter-nets’. Recently, the lower layers of the Wi-Fi protocol (IEEE 802.11 PHY/MAC layers) were adopted for higher data throughput. For very low power requirement applications, such as body-worn wireless sensors, Bluetooth also offers a low-power option [61]. However, high power consumption is still a limitation for Bluetooth, compared to other enabling technologies; in addition, since it offers ad-hoc networking, it is vulnerable to security concerns.

Another wireless enabling technology is ZigBee, built on the IEEE 802.15.4 standard, used for low power and low data rate communication. It also operates at the 2.45 GHz ISM band, with 16 channels globally; the data-rate is 250 kbps and it is capable of transmitting data between 1 to 100 metres. A data rate of 250 kbps is considered adequate for most current health applications. The digital modulation scheme is offset quadrature phase-shift keying (OQPSK). It also operates at 915 MHz in America, with ten channels at a 40 kbps data-rate; and at 868 MHz on one channel in Europe, with a 20 kbps data-rate. Star, tree and mesh topologies are all supported network architectures. Data transmission security is protected by the use of the Advanced Encryption Standard (AES) in ZigBee [62]. The battery life for ZigBee is expected to be months, whereas battery life is limited to days for Bluetooth. The target market for ZigBee is low data-rate, low energy applications. With its simple architecture and the low power requirements, ZigBee is more suited to medical applications, compared to Bluetooth.

Ultra-wide-band (UWB) technology has received attention lately as a promising method, since it offers high data rates for short ranges, as well as its very low power requirements. The US Federal Communications Commission (FCC) approved the 3.1–10.6 GHz band for UWB. UWB can provide high data rates (1 Gbps) up to a distance of ten metres. Data transmission is limited to this short range due to the low power output of UWB systems. Instead of using continuous waveforms, UWB operates with narrow pulses; therefore, the transmitters, receivers and associated amplifiers are on for very short durations. In typical indoor environments, the short-range pulses are easily detectable, which helps to prevent interference caused by the multipath effect. It is also worth mentioning that this property of UWB helps to perform position detection with high sensitivity. Finally, UWB has good penetration, so it can also be used not only for communication purposes but also for imaging [63]. Although UWB presents a promising communication method for WBANs, there are some drawbacks, such as the potential under-utilization of the allocated band due to the limited bandwidth requirements of most physiological measurement systems. In addition, the relative complexity of UWB transmitters and receivers must be overcome in a cost-effective way before UWB can compete with standards already established in the market, such as Bluetooth and ZigBee.

Other wireless standards include the medical implant communication service (MICS) band (402–405 MHz), used for bi-directional communication between the implants and body-worn/external units. This band is authorised for implantable antennas [64,65] that can be integrated into pacemakers and implantable sensors; however, there are regulatory constraints on its operation outside hospital environments that limit its usefulness in the wider WBAN market. Finally, the opening-up of spectrum in the millimetre-wave band (particularly around 60 GHz) has attracted attention for a number of short-range applications, particularly for indoor use. Although more analysis is required, due to the relatively immature nature of the field, it may offer the same advantages and disadvantages as UWB.

There is, at present, no single ‘ideal’ standard available for physiological measurement systems based on wireless body sensor networks (WBSNs) [62]. The IEEE 802.15.6 Task Group is seeking to address these issues and indications are that the new standard will have the following characteristics [62]:

- scalable data-rates, from 1 kbps to hundreds of Mbps, for use with sensors with differing bandwidth requirements;
- short-range, from two to five metres;
- network sizes of up to 100 devices will be allowed;
- it will guarantee very low latency in data paths;
- ultra-low power consumption, of the order of 0.1–1 mW, will be targeted;
- operation in the 2.36–2.40 GHz band is proposed, to take advantage of existing commercially-available technologies and mitigate potential interference.

It is unclear, at present, whether non-medical applications of wireless physiological measurement systems are envisaged for this standard, or whether it will be solely optimised for health-care applications. It is the nature of standards development that much time is required; there are many parties, both part of and exterior to the development process, who are awaiting the new standard with interest.

### 3.2. Antenna Design

Characterization of antennas is the key to establishing reliable on-body data transmission between sensors and the main data-collecting node (this may be worn on-body or an external stationary or mobile unit). Antennas for WBAN communication are required to be compact, low-weight, conformal, high efficiency and compatible with the remaining circuitry for seamless integration. Designing antennas for WBAN applications is not a straight-forward task, since antenna performance is affected by various parameters, such as impedance matching and electromagnetic absorption when placed on body. Therefore, when designing antennas for WBANs, careful consideration should be given to the presence of lossy human tissue. To this end, many antenna designs have been proposed for wearable applications, including textile antennas (*i.e.*, antennas fabricated using textile-based materials).

The effect of the presence of the human body on antenna performance has been widely investigated in the literature. In a recent study, the free-space performance of different planar antennas operating in the 2.4 GHz ISM band was analysed and compared with the on-body performance [66]. The efficiency and gain of an on-body antenna can be affected by three main parameters:

1. antenna distance from the body;
2. antenna location on the body; and
3. the antenna type.

**Figure 6.**
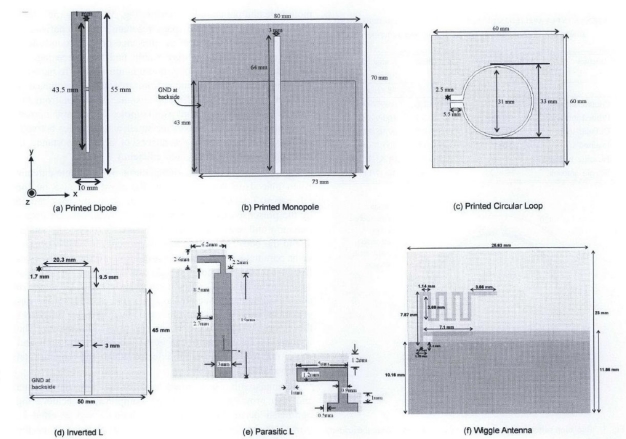
Antenna types used in [66].

In order to test the effect of the antenna’s distance from the body, six antennas (derived from the traditional dipole, monopole and loop antennas) were placed at separations of 1 mm, 4 mm and 8 mm away from the body, shown in Figure 6. It was observed that the resonant frequency was detuned as the antennas were placed closer to human body. The human body has dispersive electrical properties and is very lossy at higher frequencies; therefore, the presence of the human body changes the effective length of the antenna structure at the operating frequency of the antenna. The type of antenna is another important parameter that defines the magnitude of detuning: in the above study, it was concluded that the antennas with a ground-plane were less prone to the change in proximity to the human body, making them more suitable for WBAN applications.

The aforementioned antennas were also placed in different locations on the body, including the right and left ears, chest and ankles. According to the results, creeping waves were observed for a printed monopole antenna when placed on left chest; however, the waves decayed rapidly due to the lossy environment. Additionally, narrow-band antennas are more vulnerable to changes in the separation from the body, since even slight detuning might cause the loss of reliable data transfer. More recently, another numerical study on VHF/UHF antennas was presented [67], examining a loop, half-wavelength dipole and wideband bow-tie antenna. The effect of reflections from the ground was investigated by placing a half-wavelength dipole antenna on the leg. Reflected waves from the ground caused a multipath effect and this altered the radiation pattern and directivity, in comparison to the free-space case. Also, the reflections caused nulls at specific angular positions in the far-field patterns.

A button-shaped antenna, based on a Yagi microstrip array design and operating at 2.45 GHz, has been proposed for tele-medicine applications [68].The directivity of the antenna was enhanced with director patches, in order to prevent back-radiation and minimise losses. 8 dB bandwidth achieved at desired frequency, however, as we mentioned earlier the narrow bandwidth antennas is more likely to fail on reliable data transfer.

An innovative approach is liquid antennas for communication with implants. The electrical properties of on-body antenna substrates are very low, compared to those of the human body. The human body is mostly composed of water, which is among the materials with highest electrical properties (that is, dielectric constant and conductivity). Therefore, the near field of metal-based antennas when positioned on the skin disturbed with the reflections from body. Due to the electrical property differences, the electromagnetic waves cannot penetrate the skin and are confined in the substrate. To prevent this phenomenon and provide better electrical property matching, a liquid antenna using a saline solution was designed [69]. The liquid is encapsulated with a bio-compatible material and simulated. The liquid antenna seems a promising approach for communication with implants.

The electrical properties of the organs and skin and muscle are different; therefore, the differences in the amount of muscle or fat, or even the skin, will cause differences in the observed antenna performance. These differences may be observed with respect to gender and age differences from one subject to another; they may also be expected to vary for the same subject over time. This shows the importance of subject-specific characterisation of on-body antennas.

### 3.3. On-body Propagation

The channel variation between a transmitter and a receiver in an indoor or outdoor environment is caused by the interference between multiple scattered waves from the surrounding environment. Communication between antennas mounted on the body is achieved with both space waves (*i.e.*, propagating through the air around the body) and surface (creeping) waves (*i.e.*, propagating along the surface of the body, at the interface between air and body) [70]. The on-body propagation environment is, in some ways, similar to that in both indoor and outdoor environments, in that it is affected by scattering from the local environment. However, it differs from these propagation environments in that channel variation is affected mainly by the change in body posture. In fact, changes in the posture—hence, the geometry of the body channel—affects on-body propagation dramatically, including during even the simplest daily activities. The movement of the body parts changes the distance between antennas and causes channel variability; for example, antennas placed on a hand show dramatic channel variability [1]. Thus, on-body channel characterisation is a crucial task and needs to be studied for efficient antenna design.

**Figure 7.**
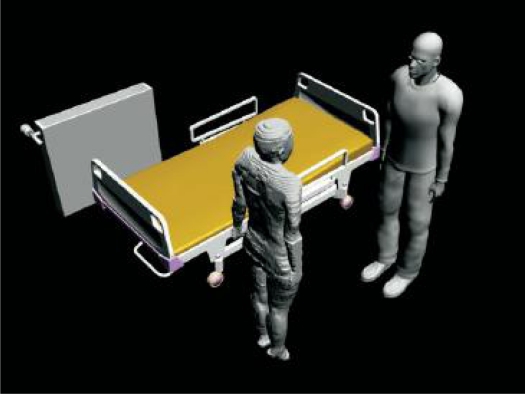
Inter-body propagation case in [71].

Two cases on-body and intra-body propagation, shown in Figure 7, have been considered in a subject-specific study [71]. For on-body propagation, a patch antenna, operating at 2.45 GHz, was placed on the left chest; the receiver was placed alternately on the front and back of the subject to investigate both the line of sight (LOS) and non-line of sight (NLOS) cases. The simulation was performed using the parallel finite difference time domain (FDTD) method and the results verified with measurements. The study concluded that the propagation along the chest was highly dependent on the chest size, where the path-loss exponent increased when the receiver and transmitter were placed on larger chests. The variability of channel for intra-body communication case is vaguely effected from change of the subjects. It has been found that the WBAN channel characterisation is subject-specific if the received signal contains creeping waves around the body.

As mentioned previously, UWB has a great potential for WBAN applications. In a recent study, the UWB radio channel was investigated experimentally with two UWB antennas [72]. Horn-shaped self-complementary (HSCA) and planar inverted cone (PICA) antennas were used for the measurement campaign. The HSCA were placed parallel to the body and the PICA were placed perpendicular to the body, in order to model the dependence of on-body channels to space-wave and surface-wave propagation. The measurements were performed with 22 different postures in an anechoic chamber. Measurement results showed that, when the antennas were placed on the chest, the HSCA demonstrated stronger surface waves than those from the PICA. Additionally, it has been shown that bi-phase modulation outperforms pulse-position modulation (PPM) with very low transmitting power and low signal-to-noise ratio (SNR). A similar recent study on UWB analysed the effect of body movement on channel parameters by using tapered slot antennas operating in the 3–9 GHz band [73]. The measurements were performed in an anechoic chamber as well as in an indoor environment; one antenna was attached to the wrist and the other antenna successively to the head, ankle, chest and back. The path-loss exponent decreased for the indoor environment, compared to that found in the anechoic chamber, due to the multipath effect. It has been observed that the received power also depends on the dynamic behaviour of the body.

### 3.4. Security and Bio-compatibility in Wireless Implantable/On-body Devices

The rapid expansion of the availability of mobile devices and wireless technologies enables the user/patient controlled usage of implantable and on-body devices. Cardiac pacemakers, insulin pumps and on-body cardiac monitoring devices are now integrated with wireless components in order to communicate with a stationary or a mobile unit, via a WBAN. Although integration of wireless technologies to such devices has many benefits, it also presents many challenges, including the enabling the secure transmission of the collected private data, prevention of electromagnetic interference between different wireless devices and compatibility with the remaining circuitry, as well as compatibility with, and safety of, the biological tissues.

Secure transmission of private medical data needs to be ensured, during both WBAN transmissions and during the submission of the data to the cloud or healthcare provider through the Internet or cellular network. Although the requirements for security and privacy of medical data are high, other constraints (such as low power requirements) on wireless health monitoring systems place limits on what can be achieved on-body. Different system architectures have been proposed in the literature. In a recent study, the security of a remote cardiac monitoring system was analysed [74]. In this paper, data transfer was modelled from the sensors to the BAN gateway, then to a wireless router and through the Internet to the monitoring server. Data collection and processing was performed in the gateway, thus memory and power requirements were high. The gateway also included two types of wireless communication: a receiver for data collection from on-body/implantable sensors and a wirless local area network (WLAN) adapter to submit the processed medical data to the wireless router. The decision unit was the monitoring server, where the collected data was profiled: if the data indicated a critical case, the unit would activate an alarm.

In [74], off-the-shelf products were used for the sensor nodes, the electrode patches, the gateway 802.11 module and the commercial server with proper storage and feedback system. The proposed wireless architecture is prone to two major security threats; the system is vulnerable during the routine usage by the patient and it is vulnerable to attacks during the transmission of the data to authorized personnel (such as the health care provider) through the web portal. During the transmission of collected data from on-body sensors, intruders could potentially attack from an unauthorized source and manipulate the vital signs data; alternatively, a denial-of-service attack could be used to prevent the collection of the sensor data by the WBAN gateway. This could cause a misinterpretation of the vital signs data and might generate a false alarm or the failure to detect a critical situation, such as a heart attack. The second vulnerability is at the link between the WBAN to the (Internet-based) monitoring server. This again could take many forms, including denial-of-service attacks and the unauthorized access to the medical data of the patient, violating privacy and confidentiality; one method might be through social engineering by targeting the healthcare providers.

Another study illustrates the privacy violation issues with different scenarios [75]. The scenarios includes the protection of the patient’s rights to protect his own healthcare record, the privacy of the healthcare provider and the restrictions on the patient to access his own information due to the purpose of the treatment. The study also illustrates the role context may have in privacy protection of both healthcare provider and patient, such as location information. The development of a secure health information acquisition and distribution architecture is vital for the reliable usage of pervasive mobile healthcare systems.

The interaction between devices emitting electromagnetic waves is highly investigated in the literature. It is well-known that such devices can cause electromagnetic interference (EMI). Recently, a study on the effect of EMI to wireless healthcare devices was performed and the possible EMI scenarios investigated [76]. An example of EMI is the interaction between high priority medical devices, such as insulin pumps, and RFID tags used in hospitals in order to identify the blood or urine samples. If the insulin pump is exposed to an RFID signal at 915 MHz, it injects the insulin without the need of it. This effect presents a high risk for diabetes patients. While there are several regulations that address the allowed levels of EMI between such devices, there are still areas where more can be done. Patients relying on implantable devices, such as insulin pumps and cardiac pacemakers, are still vulnerable to such effects.

Implantable sensors can be used either for diagnosis or sensing purposes (such as implantable antennas [77,78]), or as life support agents (such as cardiac pacemakers, drug pumps, needle-type invasive blood glucose monitoring devices and neuron stimulators). Besides the power and small size requirements, implantable devices also need to be compatible with biological tissues in order to prevent possible infection and rejection of the device by the biological tissue. Usually, implantable devices should be covered with bio-compatible materials, such as medical-grade silicon; alternatively, bio-compatible materials should be used instrinsically during the production of the implantable device. In a recent study, stens, a bio-compatible material approved by FDA, was used as a radiating structure for wireless telemetry purposes [79]. A dipole antenna was built by using stens to acquire cardiac-related data, such as blood pressure, by integrating the dipole to an implantable haemo-dynamic monitor by Medtronic. The dipole operated at 5 GHz and had a total length of 30 mm. The antenna was first simulated with HFSS, encased in a muscle ‘box’; *ex-vivo* measurements were also taken by immersing the dipole into a saline solution, simulating the electrolyte balance of the blood. Finally, *in-vivo* measurements were taken by implanting the dipole into a pig. The signals were received from the implanted stens-based dipole using a horn antenna; this proved the possibility of the usage of stens-based antennas for body telemetry.

Compared to implantable devices, on-body devices are less prone to the bio-compatibility constraint. However, long-term skin contact with such devices can cause different forms of skin irritations. A popular example of this is the previously described Ag-Cl electrode for cardiac monitoring. The adhesive gel used to ensure a good electrical contact usually causes skin irritation when exposed over a long time. Thus, on-body devices should either also be developed with bio-compatible materials, or on-body devices should be truly non-invasive, where no skin contact is required for the acquisition of the desired data (this implies the embedding of the on-body device into some wearable structure and material that can act as a protective layer to the skin).

## 4. Conclusion

In this paper, the evolution of wearable systems for chronic disease management has been discussed, with examples drawn from the literature. An evaluation of current sensing technologies has been provided for the major physiological parameters (the vital signs), as well as a review of on-body communication from an electromagnetic perspective. Current sensors technology for vital-sign monitoring is promising to alter the traditional chronic monitoring routine. However, designing non-invasive body-worn sensors is very challenging, often requiring a broad understanding of the nature of the disease and its effect on physiological parameters. Although there are sensors available off-the-shelf for cardiac and blood-pressure monitoring, there is still a need for improvement to achieve continuous and truly non-invasive monitoring of these parameters. The main constraints for sensor design are:

- low power requirements;
- reliability;
- security; and
- conformal design.

In order to achieve unobtrusive monitoring, implementation of wireless modules is vital. Integration of wireless modules to on-body sensors not only provides mobility for the patient, but also has the potential to change the conventional healthcare system with real-time feedback support. The limitations for wireless modules are low power requirements, reliable data transmission, compatibility with the sensor and conformal antenna design. Although wireless protocols are available for on-body communication, there is still a need for development, in order to ease the existing constraints. It is hoped that the efforts of the IEEE 802.15.6 Task Group will bear fruit in this area.

Although the design and implementation of on-body monitoring systems presents a challenging task with several constraints, the benefits of employing multi-parameter monitoring systems for the prevention, prediction and management of diseases are myriad. On-body monitoring systems with multiple sensors are not only capable of providing an extensive database of the patient’s medical history: the simultaneous usage of multi-parameter monitoring sensors can also verify or correct the collected data, adding redundancy into a potentially safety-critical system; or the additional information can place a particular event detected into context. For example, the previously mentioned blood pressure monitoring system in [13] can be used in combination with ECG monitoring device for acquired data verification purposes: if there is a failure in the electronic circuitry or software of the cardiac-activity monitoring system, a critical situation can be triggered by the blood pressure monitoring device. Another example where a multi-parameter monitoring system could bring benefits is the combination of a blood-pressure monitor and blood-glucose level monitor: the BP monitor could provide context (in the form of blood flow rate differences) for the calculation of BGL by the BGL sensor, leading to improvements in the accuracy of the measurement(The authors are grateful to the reviewer for suggesting this example). On the other hand, there may be alternative interpretations of certain symptoms (that is, there may be more than one cause) that could be more easily resolved given more information; for instance, a (sudden) drop in blood-sugar levels would be of more concern if the patient had been relatively inactive, compared to if the system had detected an increase in physical activity. Thus, multi-parameter monitoring systems will be more reliable and useful, compared to single-parameter monitoring devices.

Finally, over recent years our group has contributed to the literature on this topic, with numerous publications on the characterisation of on-body channels, the investigation of the effect of the human body on antenna performance, as well as the establishment of a communication link between sensors and body-worn units. Our goal is to utilize the accumulated knowledge within the group on on-body propagation to design multi-parameter body-worn units. To this end, some of the current research activities in our group focus on non-invasive microwave detection of blood glucose levels and reliable transmission of ECG signals.
